# Inhibition mechanism of CDK-2 and GSK-3β by a sulfamoylphenyl derivative of indoline—a molecular dynamics study

**DOI:** 10.1007/s00894-017-3395-8

**Published:** 2017-07-19

**Authors:** Przemysław Czeleń

**Affiliations:** Department of Physical Chemistry, Faculty of Pharmacy, Collegium Medicum, Nicolaus Copernicus University, Kurpinskiego 5, 85-096 Bydgoszcz, Poland

**Keywords:** CDK-2, GSK-3β, Docking, Molecular dynamics, MMPBSA, Selective inhibition

## Abstract

A good understanding of the inhibition mechanism of enzymes exhibiting high levels of similarity is the first step to the discovery of new drugs with selective potential. Examples of such proteins include glycogen synthase kinase-3 (GSK-3β) and cyclin-dependent kinase 2 (CDK-2). This article reports the mechanism of such enzyme inhibition as analyzed by an indoline sulfamylophenyl derivative (CHEMBL410072). Previous work has shown that such compounds exhibit selective properties towards their biological targets. This study used a combined procedure involving docking and molecular dynamics simulations, which allowed identification of interactions responsible for stabilization of complexes, and analysis of the dynamic stability of the systems obtained. The initial data obtained during the molecular docking stage show that the ligand molecule exhibits a similar affinity towards both active sites, which was confirmed by quantification of identified interactions and energy values. However, the data do not cover dynamic aspects of the considered systems. Molecular dynamics simulations realized for both complexes indicate significant dissimilarities in dynamics properties of both side chains of the considered ligands, especially in the case of the part containing the sulfamide group. Such increased mobility of the analyzed systems disrupts the stability of binding in the stabilized complex with GSK-3β protein, which finally affects also the binding affinity of the ligand molecule towards this enzyme.

## Introduction

The most important role in regulation and control of all cellular processes is played by proteins belonging to the group “kinases”. The numerous applications of such enzymes are due to the significant structural polymorphism of these systems. However, this does not refer to the active site responsible for binding the ATP molecule. Indeed, high similarity is observed in the amino acid sequences creating the binding site of this group of enzymes, which significantly hinders the discovery of new selective drugs dedicated to specific biological targets. Examples of such proteins include glycogen synthase kinase-3 (GSK-3β) [1] and cyclin-dependent kinase 2 (CDK-2) [2]; GSK-3β could be classified as a serine/threonine protein kinase, and its primary activity is the inactivation of glycogen synthase carried out by direct phosphorylation [3–5]. GSK-3β activity has also been found during other cellular processes associated with β-catenin degradation [6], protein translation [7] and maintaining of microtubule stability [1, 8]. Disorders in the proper functioning of GSK-3β are related to the occurrence of many diseases, such as Alzheimer’s disease [8], diabetes [9, 10], renal proliferative diseases [11] and cancers [10]. The main activity of serine/threonine kinase (CDK-2) involves regulation of the proliferation of eukaryotic cells. The significant CDK-2 activity in processes related to control of the cell division cycle is responsible for the fact that all enzymes from CDKs family are important therapeutic targets [12–14]. The main role of the CDK-2 is in regulation of the G1 phase of the cell cycle. Disorders in the proper functioning of this enzyme are related with the occurrence of many cancer diseases [15–19]. As noted, both these kinases are involved in many different cellular processes, and their dysfunction is associated with numerous diseases; however, the high similarity of their active sites [20] significantly hampers the discovery of new compounds exhibiting selective potential towards these two biological targets. A large group of ATP-competitive inhibitors of CDK-2 and GSK-3β characterized by similar affinity is known [21, 22], while there is a much smaller group of chemical compounds with confirmed selective potential towards these two enzymes, for example indirubine derivatives and analogs [2, 23–29]. Previous work has also shown that the sulfonyl derivative of indoline can exhibit selective properties in the context of inhibition of these two proteins [30]. A better understanding of the mechanism of interaction of such inhibitors with the active site will allow the development of new compounds.

## Methods

### Docking procedure

**Fig. 1:**
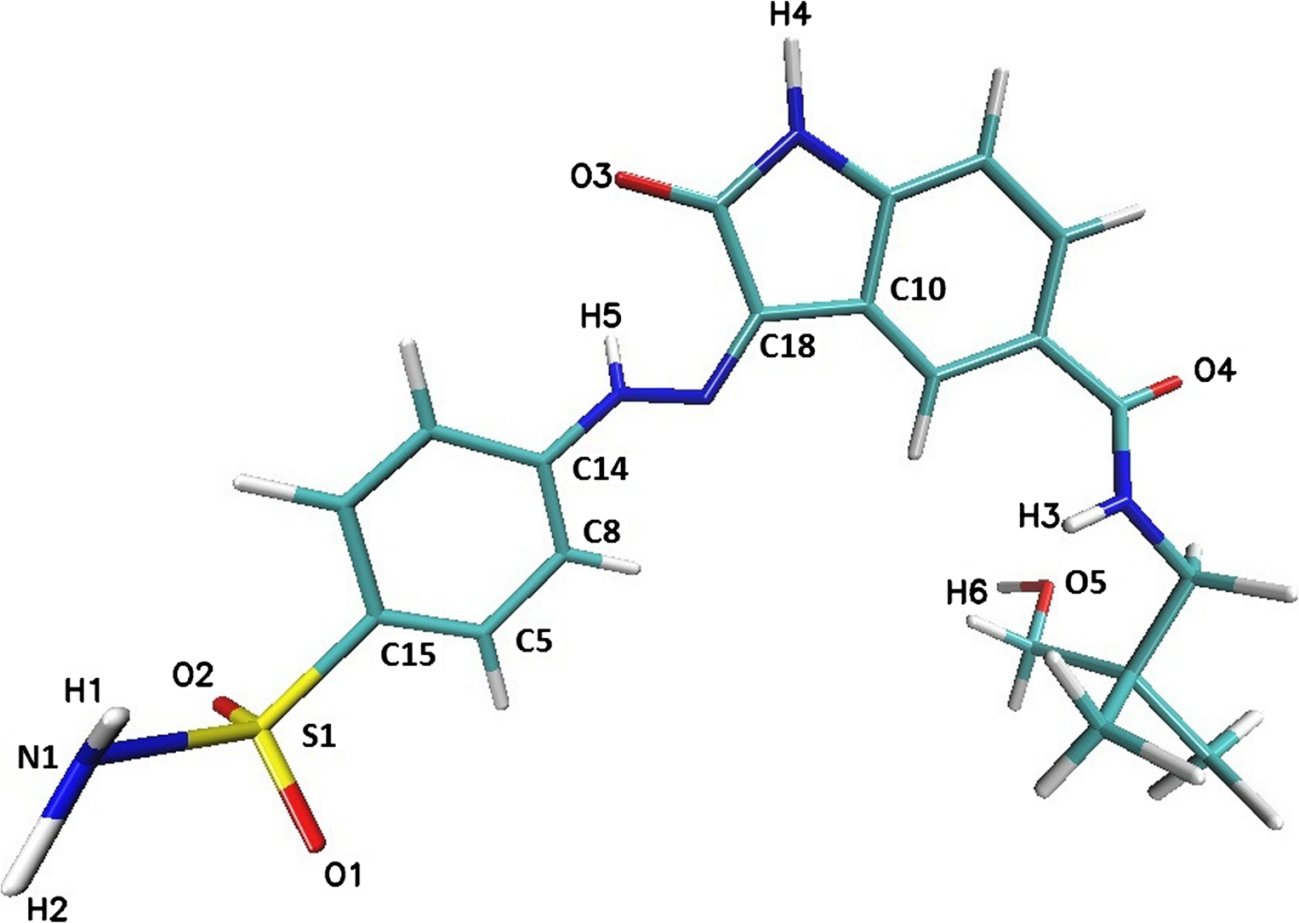
Graphical representation of ligand molecule structure (CHEMBL410072)

Proteins used during the docking procedure, namely CDK-2 (PDB ID 1E9H) [2] and GSK-3β (PDB ID: 1Q41) [1], were downloaded from Brookhaven Protein Database PDB. The ligand molecule LIG (Fig. 1) (CHEMBL ID: CHEMBL410072) (3Z)-N-(3-hydroxy-2,2-dimethylpropyl)-2-oxo-3-[(4-sulfamoylphenyl)hydrazono]-5-indolinecarboxamide] was obtained from the CHEMBL Database. This stage of modeling was realized with the use of AutoDockVina [31] based on united-atom scoring function. Enzyme structures without any water molecules were used during calculations; also, all nonpolar hydrogen atoms were removed from the considered molecules. The dimensions of the grid box for both active sites were specified by the following sets of values: 16 × 14 × 22 for CDK-2 and 22 × 22 × 16 for GSK-3β. All preliminary steps were realized with the help of the Auto Dock Tools package. For each considered system, the docking procedure was realized ten times, which provided a sufficient population of conformations characterized by the most favorable values of binding affinity. From all collected structures, in the molecular dynamics stage, conformations that provides occurrence of interactions with key amino acids from the active sites of the CDK-2 and GSK-3β enzymes were chosen.

### Molecular dynamics simulation

**Fig. 2:**
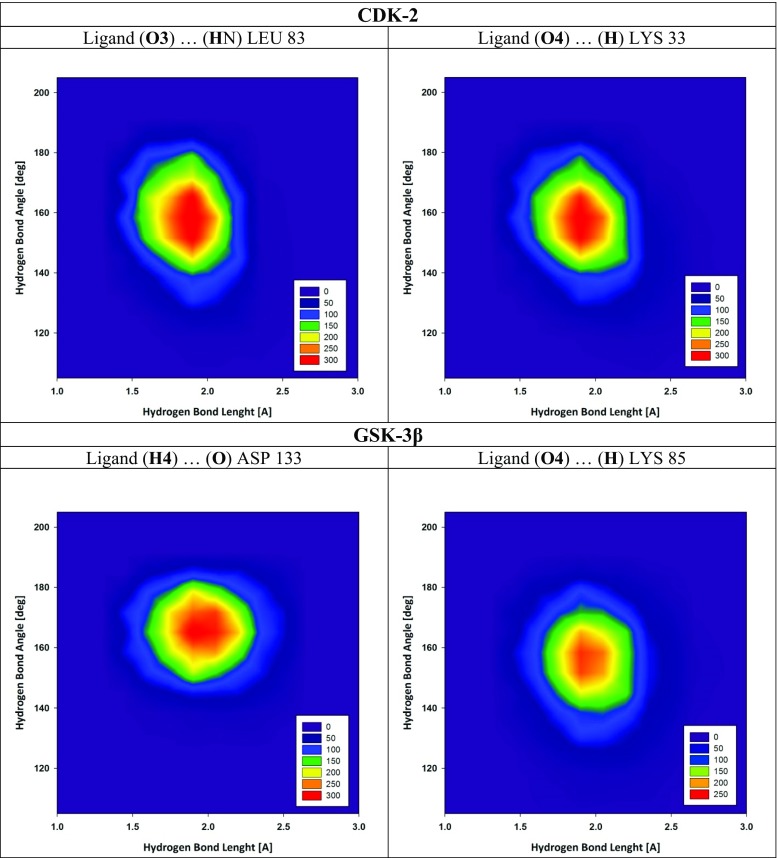
Mutual distribution of hydrogen bonds lengths and angles for most important interactions observed in active sites of cyclin-dependent kinase 2 (CDK2) and glycogen synthase kinase-3 (GSK-3β) proteins

All molecules used during the molecular dynamics (MD) simulations stage were characterized using the Amber Force Fields. In the case of both proteins (CDK-2 and GSK-3β) the ff99SB Force Field [32] was used, while the structure of the ligand molecule was described using Gaff Force Field [33] and the atomic charges were calculated via the RESP procedure [34] at HF/6-31G* level of theory. The structures of complexes obtained during docking stage were neutralized and drenched by a periodic box of TIP3P water molecules. Both systems were minimized, and two stages of this process were realized with the use of steepest descent and conjugate gradient methods. After minimization, each system was heated up to 300 K by 100 ps of initial MD simulation. The temperature of the systems was controlled by the Langevin thermostat [35]. The 100 ns of MD simulation were realized in periodic boundary conditions with the SHAKE algorithm [36]. In both systems, after considering root mean square deviation (RMSD) values distributions, the first 20 ns of the simulations were treated as an equilibration stage, while the next 80 ns of simulation were used in the analysis of properties of complexes. The binding affinity of the ligand towards the proteins was evaluated by the Molecular Mechanics /Poisson Boltzmann Surface Area method (MM/PBSA) [37]. In the MM/PBSA calculations, the polar desolvation free energy was calculated by PB solver implemented in the PBSA module [38]. During calculations realized in the TIP3P explicit solvent, the atomic radii optimized by Tan and Luo were used [39]. All MD simulations were performed using the AMBER 11 package [40]. The structural analyses, which include evaluation of hydrogen bonds, distances, dihedral angles and RMSD of atomic position, were realized using the VMD package [41]. During analysis, hydrogen bonds were defined by the following criteria: distance between donor (D) and acceptor (A) < 3.5 Å, angle D–H–A > 90°, and distance H–A < 3 Å. The geometric criteria defining hydrogen bond interactions included two factors: the distance between hydrogen and acceptor atoms; and the value of valence angle created by donor, hydrogen and acceptor atoms. The strength of such interactions is strictly related with both geometric factors mentioned. Figure 2 presents the mutual distributions of hydrogen bond distances and angles for chosen interactions observed in the active sites of both proteins. In all cases, we can observe that, for all fractions of hydrogen bonds lengths, the width of angles values distributions does not exceed 40°, and the majority of considered conformers is described by values in the range of 20°. The similarity of distributions of angle values for all fractions regardless of bond length led to distance between hydrogen and acceptor atom being chosen as the descriptor of strength of hydrogen bond interactions in this work. The RMSD calculations performed for the systems considered were realized relative to the initial structure of complexes obtained during the docking stage; during these calculations all hydrogen atoms were excluded.

## Results

### Docking

**Fig. 3:**
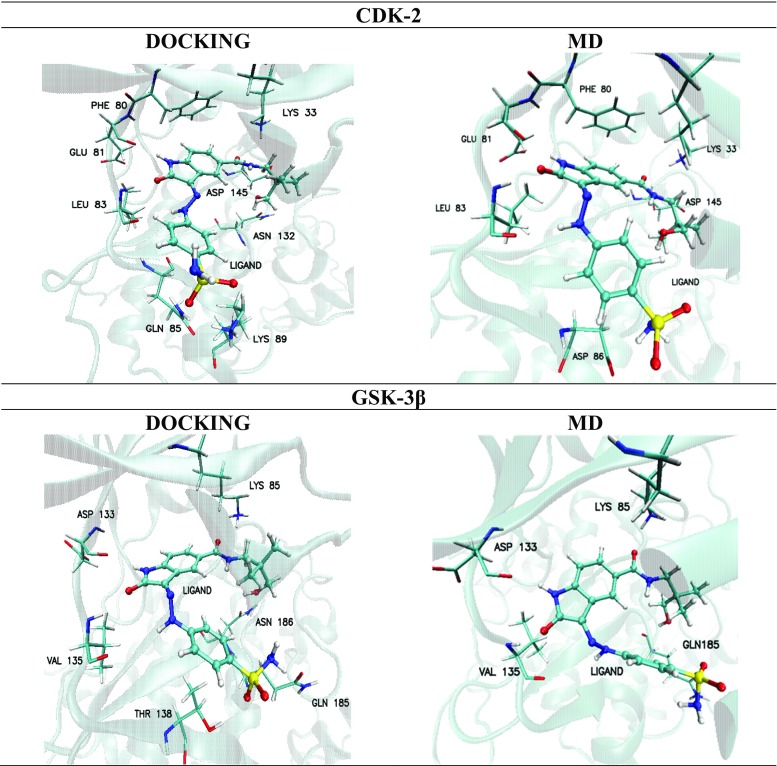
Graphic representation of the most important amino acids involved in creation of interactions identified during docking procedure and molecular dynamics (MD) stage

The structures of complexes obtained during the docking stage were stabilized by numerous interactions of different types. One of the most important contributions to the stabilization of the considered complexes was represented by hydrogen bond interactions. The criterion for classification of the strength of such interactions is assessment of the distance between acceptor and hydrogen atoms: strong interactions are characterized by a distance <1.6 Å, medium strength by values in the range from 1.6 Å to 2.0 Å, and weak by distances >2 Å. In the structure of the complex created by the CDK-2 protein with the ligand molecule, eight interactions with different strengths were identified (Fig. 3). The indoline core of the ligand molecule creates two types of hydrogen bonds with glutamic acid GLU81 and leucine LEU83, and these two interactions are characterized by the shortest distances between donors and acceptors of hydrogen bonds. The oxygen O4 of the amide group creates two hydrogen bonds: one with the hydrogen from the amino group of lysine LYS33, and the second with the amide proton from aspartic acid ASP 145. The distances between the atoms clearly show that the interaction with lysine is much stronger. The hydroxyl group localized on the end of the side chain of the ligand is involved in creation of a medium strength hydrogen bond with the carbonyl oxygen atom from the side chain of asparagine ASN132. The second side chain of the ligand molecule also creates important interactions, namely hydrogen H5 interacts with the carbonyl oxygen from LEU83; likewise, both oxygens from the sulfamide group are involved in creation of hydrogen bonds with the amino groups of glutamine GLN85 and lysine LYS89. The orientation of the aromatic ring of phenylalanine PHE80 relative to the indoline core of the ligand allows for the occurrence of stacking interactions. The presented interactions identified during the docking stage confirm previous investigations that had indicated the important role played by GLU81, LEU83 and LYS 33 in creation of interactions with ligands in the active site of the CDK-2 enzyme [2, 29, 30].

The complex of GSK-3β with the ligand molecule obtained during the docking stage was stabilized by six hydrogen bonds. The indoline core of the ligand molecule, as in earlier analyzed complexes, creates two interactions, namely with aspartic acid ASP133 and valine VAL135. The distances between interacting atoms creating these bonds indicate the highest stability of all observed interactions in the complex. The participation of the same amino acids in the formation of hydrogen bonds describing the mechanism of GSK-3β protein inhibition is also confirmed by the research literature [1, 29, 30]. Oxygen O4 of the amide group is involved in creation of a hydrogen bond with hydrogen atoms from the amino group of lysine LYS85. The hydrogen atom from the hydroxyl group creates an interaction with the carbonyl oxygen from the side chain of asparagine ASN186.

The next contribution to stabilization of the considered complex comes from interactions of atoms from the sulfamide group, namely oxygen O1 with the hydroxyl group of threonine THR138, and from the amide group, namely hydrogen with the carbonyl oxygen from the side chain of glutamine GLN185. The binding affinities of ligand molecule towards active sites in the considered complexes are quite similar, −9.30 (±0.13) kcal mol^−1^ for CDK-2 and -9.20 (±0.15) kcal mol^−1^ for GSK-3β.

### Molecular dynamics

#### Structural and dynamic stability of complexes

**Fig. 4a–d:**
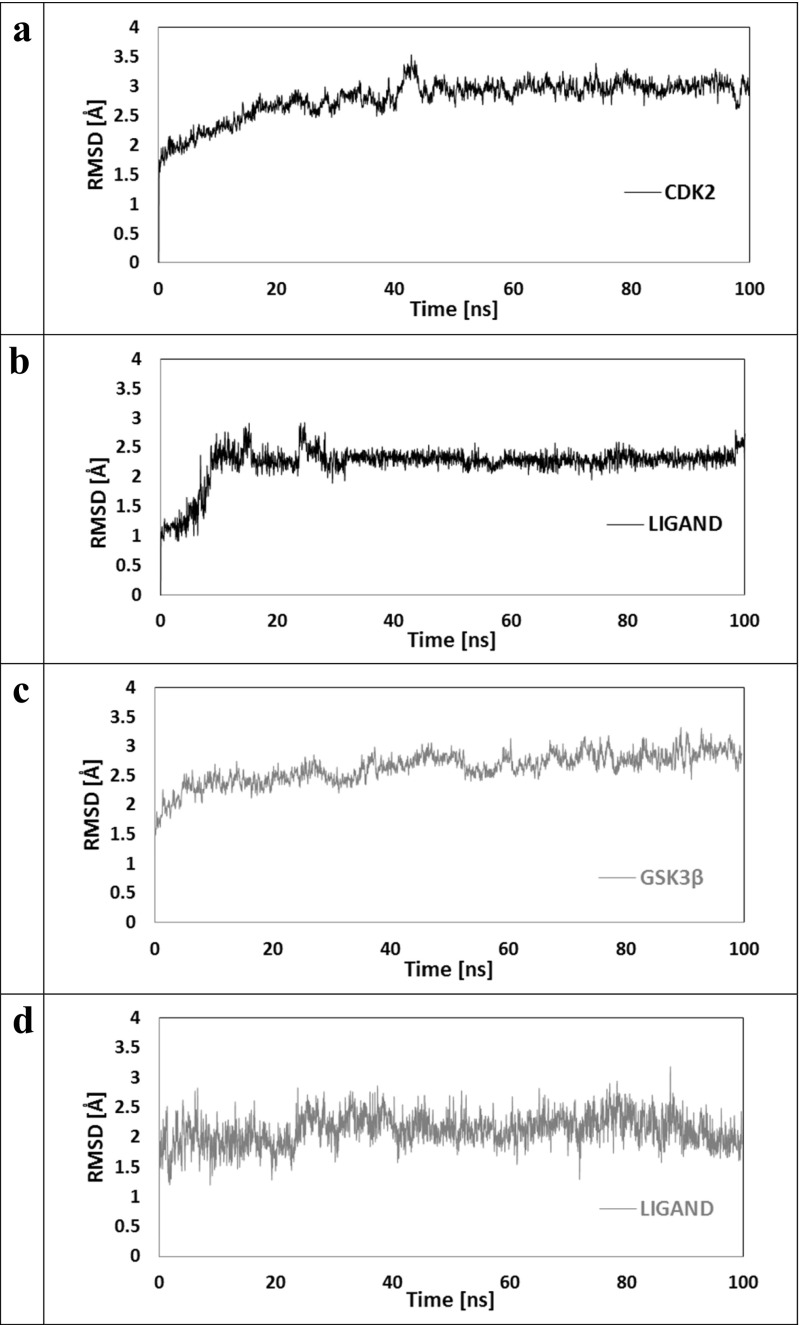
Distribution of root mean square deviation (RMSD) values. **a**, **b** *Black* Subunits creating ligand–CDK-2 protein complex. **c**, **d** *Gray* Subunits creating ligand–GSK-3β complex

The most representative structures of complexes obtained during the docking stage were used as starting points for MD simulations. The structural and dynamic stabilities of the considered systems were evaluated from RMSD values, which were calculated for each enzyme and ligand molecule. The time evolution of these values is presented in Fig. 4. It can be observed that a 20-ns equilibration period is sufficient for all considered structures. Averaged values characterizing all conformations used during structural analysis are noted in Table 1. All general values describing both enzymes used during this investigation indicate quite similar global mobility of amino acids creating both active sites. Averaged values describing ligands in both complexes reveal an interesting dependence. Higher RMSD values describing changes in conformation of the ligand in complex with the CDK-2 protein indicate more significant changes relative to the docking structure compared with the complex formed from GSK-3β. However, the two times smaller standard deviation (SD) values indicate the higher conformational stability of ligands in the first complex compared with the second.

**Table 1:** Averaged values of root mean square deviation (RMSD) and standard deviation (SD) for ligand and enzymes for all steps used during structural analysis

	Ligand [CDK-2]	CDK-2	Ligand [GSK-3β]	GSK-3β
RMSD	2.92	2.31	2.72	2.17
SD	0.16	0.12	0.19	0.24

**Fig. 5a,b:**
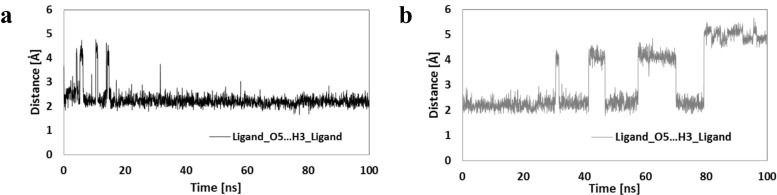
Distributions presenting time evolution of the internal hydrogen bond between oxygen O5 and the hydrogen atom H3.** a** Ligand bound to CDK-2 protein.** b** Ligand bound to GSK-3β

**Fig. 6a–d:**
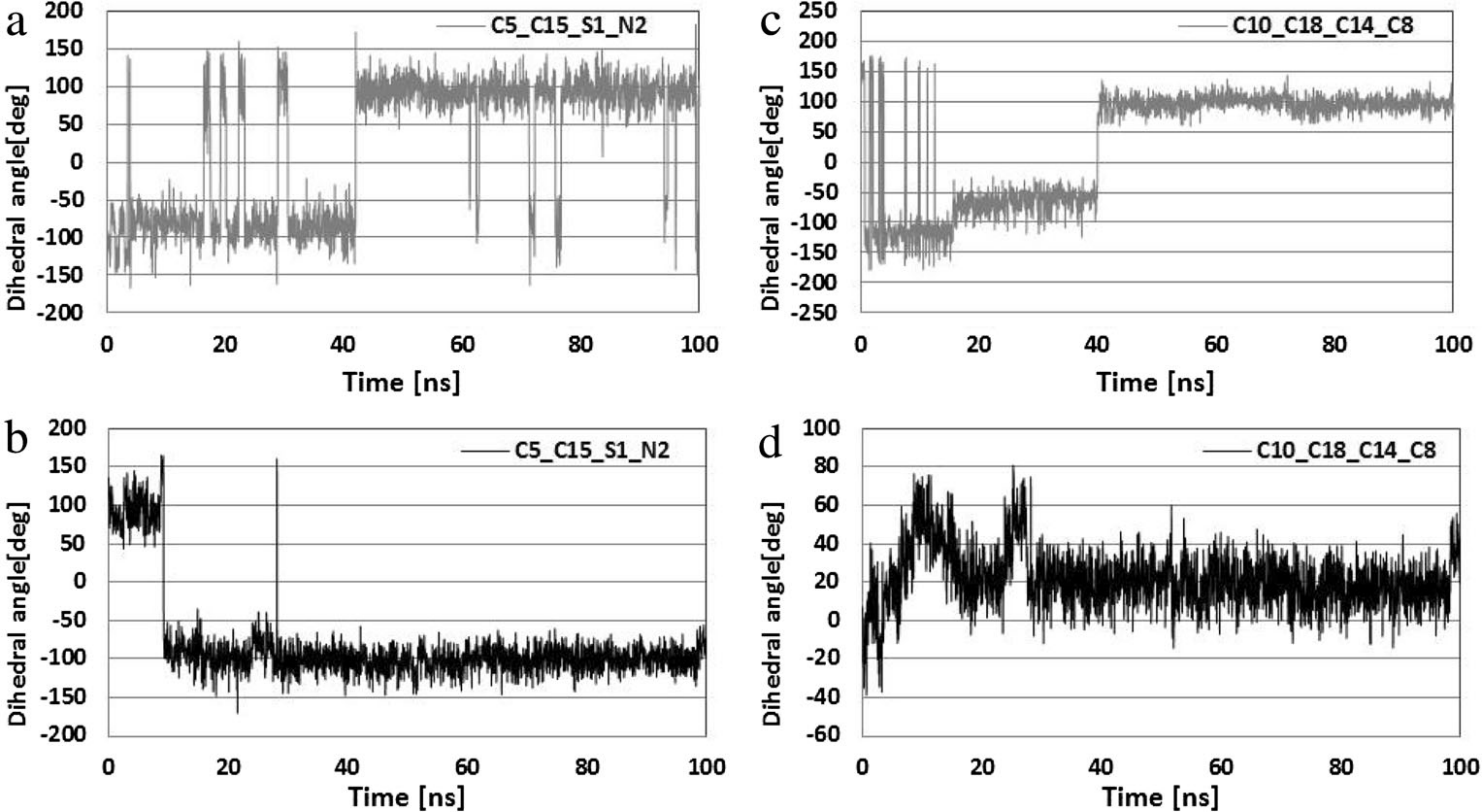
Distribution of dihedral angle (deg) describing conformational changes in side chains containing a sulfamide group.** a**,** c** Distributions represent values describing ligand bound to GSK-3β.** b**,** d** Distributions represent ligand bound to CDK-2 protein

The conformational changes of the ligand molecule in both complexes are mainly related to the rotations of their side chains. In both initial conformations obtained during docking stage, the orientation of the side chain containing the hydroxyl group supports the creation of an internal hydrogen bond between amide hydrogen H3 and oxygen O5. Figure 5 presents distributions describing evaluation of the distances between both atoms. These data clearly show a weak hydrogen bond created between the ligand and the CDK-2 protein observed during the whole MD simulation time, while in the case of ligand coming from the second complex numerous fluctuations were observed (∼40% simulation time), which indicate the increased rotational mobility of this part of the ligand molecule. The conformational changes in the second side chain were described with the use of dihedral angle values; all atoms used in the definition are marked in Fig. 1. The rotation of the sulfamide group was defined using the dihedral angle C5–C15–S1–N2 and its distributions are presented in Fig. 6a,b. Two comparable populations of values were observed for the ligand structure bound to GSK-3β protein, indicating the presence of two competitive conformations of the sulfamide group. As evidenced by occasional changes in orientation, the obtained configuration is not stable. In the case of the ligand forming a complex with the second protein, the initial conformation was changed after 10 ns of simulation time to the opposite orientation, and this was maintained during the remaining simulation time without any fluctuations. The mutual orientation of phenyl ring and indoline core of ligand was described by values of dihedral angle C10–C18–C14–C8 (Fig. 6c,d).

The fluctuations in values observed for the ligand-forming complex with the GSK-3β enzyme show that, in the first phase of MD, this fragment of the molecule underwent numerous conformational changes. The final value of this parameter was determined after 40 ns of MD simulation time. In the case of complex ligand-CDK-2, slight fluctuations in the dihedral angle C10–C18–C14–C8 were observed during the equilibration stage, and after this period its value was stabilized at about 20°, which does not differ significantly from initial value (7°).

#### Interactions stabilizing ligand complexes with CDK-2 and GSK-3β proteins

Stability of ligand complexes with both proteins, namely CDK-2 and GSK-3β, is strictly related with interactions occurring in their active sites. The time evolution of the analyzed systems realized during MD simulations allowed evaluation of the stability of all bindings observed during the docking stage, and the finding of new, potentially important, effects related to the conformational changes of the ligand molecule and enzyme binding pocket. The complex with CDK-2 is stabilized by several interactions. The most stable interactions were found for oxygen O5 and hydrogen H4 from the indoline core of the ligand molecule. These atoms participate in the creation of hydrogen bonds with leucine LEU83 and glutamic acid GLU81, respectively, and both were observed during the whole simulation time, as confirmed by data presented in Table 2. Of all the hydrogen bonds observed, the most stable interaction is Ligand(O3)⋯(HN)LEU 83, and nearly 97% of these impacts can be classified as medium strength with length fluctuating in the range from 1.75 Å to 2 Å. The second interaction, namely [Ligand(H4)⋯(O)GLU81] is characterized by very similar length values of hydrogen bonds. In this case, 94% of conformers create bonds with medium strength. Such observations indicate the very slight mobility of the ligand core in the conformational space of the active site. The hydrogen bond formed between the H5 atom from the side chain of the ligand molecule and the carbonyl oxygen of LEU 83 also seems to be an important interaction. This interaction is more labile than the two prior bonds, which was observed in 87% of the collected conformations after MD simulation. Oxygen O4 of the amide group also plays an important role in the stability of complex, creating two possible bonds. The significant interaction is created with hydrogen atoms from the amino group of lysine LYS33, and it is observed over nearly the entire simulation time. Over 87% of such interactions could be characterized as medium strength hydrogen bonds. For 30% of conformations, a second, more labile interaction was observed, with peptide hydrogen atoms belonging to aspartic acid ASP145. The last hydrogen bond created by the considered ligand was observed between the hydrogen atom from the sulfamide group and oxygen atoms from carboxyl group from the side chain of aspartic acid [Ligand(NH_X_)⋯(O)ASP 86]. This interaction was not observed in the structure of the complex obtained during the docking stage. Its origin is related to rotation of the sulfamide group relative to the phenyl ring, which enabled the occurrence of that hydrogen bond. This interaction was found in 91% of conformations collected during MD simulation. Most of them (82%) are medium strength hydrogen bonds. It is worth noting that, after final formation of this interaction, its length does not fluctuate and is in the range from 1.75 Å to 2 Å.

**Fig. 7:**
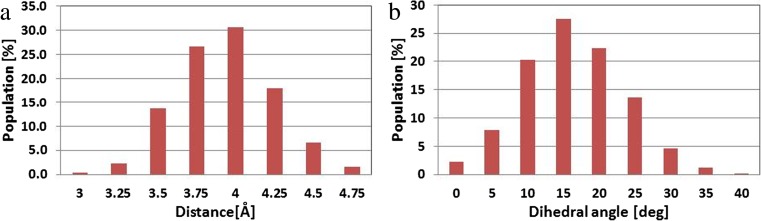
**a**,**b** Histograms presenting distribution of distances between aromatic systems of phenylalanine PHE 80 and the ligand molecule (**a**), and dihedral angles describing mutual orientation of planes of aromatic systems of considered molecules (**b**)

It is important to state that not all the hydrogen bonds identified during the docking stage were observed in the conformations collected during MD simulations. Such a situation applies to interactions with glutamine GLN85, lysine LYS89 and asparagine ASN132. In the case of interactions with the former two amino acids, the bonds were created with amino groups from the side chains. Both of these were localized on the edge of the binding active site and were characterized by significant lability. Rotation of the sulfonamide group contributes to the instability of these interactions. Another type of interaction was observed in the case of phenylalanine PHE80. The orientation and distance between aromatic systems of this amino acid and the ligand molecule allows for the occurrence of stacking interactions. The distributions presented in Fig. 7 describe the mutual orientation of these molecules. In 95% of these conformations the distance between aromatic rings does not exceed 4.5 Å. Their mutual mobility is also limited, which confirms distributions of the dihedral angle describing the mutual rotation of the aromatic systems considered. The GSK-3β–ligand complex is maintained by four hydrogen bonds. By analogy with the case of CDK-2 protein, the most important interactions are created by atoms from the indoline core of molecule. The first of them is created by hydrogen atom H4, with a carbonyl oxygen from aspartic acid ASP 133 [Ligand(H4)⋯(O)ASP133]. This interaction is quite stabile during the whole simulation. Over 74% of conformations create hydrogen bonds with medium strength. The second interaction localized in the core of ligand molecule is the bond created by oxygen O3 and hydrogen atom from peptide group of valine VAL135 [Ligand(O3)⋯(H)VAL135]. This bond was observed in the analogous position to the previous one during the whole simulation. About 72% of these interactions could be classified as medium hydrogen bonds. The hydrogen bond created by oxygen O4 and hydrogens from the amino group of leucine LEU85 also seems to be important. This bond could be found in 87% of conformations collected during MD simulation. Even so, for 67% of conformers, a decrease in the share of the strongest interactions could also be observed. The final interaction found during MD simulations is the bond created with the glutamine GLN185 [Ligand(OH)⋯(O) GLN185]. This hydrogen bond was found only in 67% of conformers. The largest share in the analyzed population was weak interactions. Among all the interactions observed during docking stage, those involving side chains containing a sulfamide group of ligand proved to be unstable during MD simulation. The significant lability of this part of the ligand molecule precludes the creation of stable interactions. Structural properties of the considered complexes are correlated strictly with the affinity of the ligand molecule for both active sites. Calculations of enthalpic and entropic contributions to Gibbs free energy were performed to allow quantitative evaluation of both interactions. All values presented in Table 3 unambiguously show that these ligand molecules exhibit higher binding affinity towards the CDK-2 active site. The differences in the enthalpy values intensify if entropic contributions to Gibbs free energy are included, which is caused by the fact that the binding affinity of ligand molecule towards the CDK-2 enzyme is twofold higher compared with the second protein.

**Table 2:** Distribution of the most frequently created hydrogen bonds between ligand molecule and selected amino acids from CDK-2 and GSK-3β active sites

Hydrogen bond	Length of hydrogen bond [Å]^a^	Population %	
CDK-2			
Ligand (H4)⋯(O) GLU 81	1.75	44.2	∼ 100
	2	50.1	
	2.25	5.4	
	2.5	0.2	
Ligand (O3)⋯(HN) LEU 83	1.75	51.8	∼ 100
	2	44.9	
	2.25	2.9	
	2.5	0.3	
Ligand (H5)⋯(O) LEU 83	1.75	1.3	∼ 87.5
	2	14.4	
	2.25	24.4	
	2.5	22.6	
	2.75	16.2	
	3	8.7	
Ligand (O4)⋯(H) LYS 33	1.75	39.6	∼ 99.9
	2	47.9	
	2.25	10.2	
	2.5	2.1	
Ligand (NH_X_)⋯(O) ASP 86	1.5	0.6	∼ 91
	1.75	46.3	
	2	36.3	
	2.25	6.1	
	2.5	1.4	
	2.75	0.4	
GSK-3β			
Ligand (H4)⋯(O) ASP 133	1.5	0.2	∼ 100
	1.75	34.4	
	2	39.5	
	2.25	25.5	
	2.5	0.4	
	2.75	0.0	
Ligand (O3)⋯(H)VAL 135	1.75	24.3	∼ 100
	2	48.3	
	2.25	25.1	
	2.5	2.1	
	2.75	0.2	
Ligand (H5)⋯(O)VAL 135	2	1.4	∼ 10
	2.25	1.7	
	2.5	1.8	
	2.75	2.3	
	3	2.8	
Ligand (O4)⋯(H) LYS 85	1.75	25.7	∼ 87.4
	2	42.0	
	2.25	17.5	
	2.5	1.8	
	2.75	0.2	
	3	0.2	
Ligand (OH)⋯(O) GLN 185	1.75	8.4	∼ 67.7
	2	21.0	
	2.25	17.3	
	2.5	10.4	
	2.75	6.3	
	3	4.1	

^a^The hydrogen bonds in the table represent middle values of intervals with width of 0.25 Å

**Table 3:** Enthalpic and entropic contribution to binding free energy values (Δ*E* kcal mol^−1^ with SD) for the CDK-2 and GSK-3β complexes with ligand observed during MD simulations

	CDK2		GSK-3β	
	∆*E*	SD	∆*E*	SD
*E*_VDWAALS_^a^	−54.07	3.64	−47.89	3.95
* E*_EL_^b^	−59.71	8.99	−39.29	7.65
* E*_PB_^c^	78.50	9.11	60.23	7.56
*E*_CAVITY_^d^	−5.04	0.14	−3.77	0.18
∆* H*_PB_	−40.31	5.34	−30.71	4.74
TΔ* S*	−16.57	4.87	−18.87	3.25
Δ* G*_PB_	−23.74	7.22	−11.86	5.75

^a^van der Waals contribution calculated by the MM force field

^b^Electrostatic energy as calculated by the MM force field

^c^Electrostatic contribution to the solvation free energy calculated by PB

^d^Nonpolar contribution to the solvation free energy

## Conclusions

Both proteins considered in this article exhibit high similarity in terms of the structure of the active site, as confirmed by values obtained during docking procedures. Similar numbers of hydrogen bonds and nearly identical characteristic of the bonds created by the indoline core of the ligand could indicate nearly identical binding properties of the ligands considered relative to both proteins. However, this analysis does not take into account the dynamic properties of ligand and amino acids forming the enzyme active sites. Time evolution analysis of the considered systems unambiguously shows that this aspect has a significant impact on ligand binding affinity towards both compared enzymes. Increased rotational mobility of the side chains of the ligand molecule in complex with GSK-3β significantly impedes the creation of stable interactions. In the case of all hydrogen bond interactions identified during the docking stage for atoms incorporated in the side chains of the ligand, disappearance of these interactions (THR138 and ASN186) or their significant weakening (GLN185) was observed. Also, in the case of the more stable interactions created with ASP133, VAL135 and LYS85, a noticeable weakening was observed relative to analogical bindings observed in the complex with CDK-2, as manifested in a reduction of the number of interactions and general increase in the quantity of weak hydrogen bonds. Ligands bound with CDK2 protein create five stable hydrogen bonds, which were observed over nearly the entire simulation time; most of these could be classified as the strongest interactions. Stacking interactions with phenylalanine PHE80 represent an additional factor increasing the affinity of the ligand molecule for the CDK-2 binding site. All structural relations observed for the complexes considered here influence the general values of binding affinity of the ligand molecule towards both active sites. Increased mobility of the ligand in the GSK-3B binding site, and the general weakening of specific interactions caused a significant decrease of Gibbs free energy value, which was twofold smaller than for the second complex. Such discrepancies in structural and energetic properties of the considered complexes could indicate a potential selectivity of the considered ligand in the context of interactions with both proteins used.
